# Update to a protocol for a feasibility cluster randomised controlled trial of a peer-led school-based intervention to increase the physical activity of adolescent girls (PLAN-A)

**DOI:** 10.1186/s40814-016-0110-y

**Published:** 2016-12-01

**Authors:** Simon J. Sebire, Mark J. Edwards, Rona Campbell, Russell Jago, Ruth Kipping, Kathryn Banfield, Bryar Kadir, Kirsty Garfield, Ronan A. Lyons, Peter S. Blair, William Hollingworth

**Affiliations:** 1 Centre for Exercise, Nutrition & Health Sciences, School for Policy Studies, University of Bristol, Bristol, UK; 2 School of Social & Community Medicine, University of Bristol, Bristol, UK; 3 Farr Institute, Swansea University Medical School, Swansea, UK

**Keywords:** Physical activity, Peers, Adolescent girls, Intervention, School

## Abstract

**Background:**

Physical activity levels are low amongst adolescent girls, and this population faces specific barriers to being active. Peer influences on health behaviours are important in adolescence, and peer-led interventions might hold promise to change behaviour. This paper describes the protocol for a feasibility cluster randomised controlled trial of Peer-Led physical Activity iNtervention for Adolescent girls (PLAN-A), a peer-led intervention aimed at increasing adolescent girls’ physical activity levels. In addition, this paper describes an update that has been made to the protocol for the PLAN-A feasibility cluster randomised controlled trial.

**Methods/design:**

A two-arm cluster randomised feasibility trial will be conducted in six secondary schools (intervention *n* = 4; control *n* = 2) with year 8 (12–13 years old) girls. The intervention will operate at a year group level and consist of year 8 girls nominating influential peers within their year group to become peer supporters. Approximately 15% of the cohort will receive 3 days of training about physical activity and interpersonal communication skills. Peer supporters will then informally diffuse messages about physical activity amongst their friends for 10 weeks. Data will be collected at baseline (time 0 (T0)), immediately after the intervention (time 1 (T1)) and 12 months after baseline measures (time 2 (T2)). In this feasibility trial, the primary interest is in the recruitment of schools and participants (both year 8 girls and peer supporters), delivery and receipt of the intervention, data provision rates and identifying the cost categories for future economic analysis. Physical activity will be assessed using 7-day accelerometry, with the likely primary outcome in a fully powered trial being daily minutes of moderate-to-vigorous physical activity. Participants will also complete psychosocial questionnaires at each time point: assessing motivation, self-esteem and peer physical activity norms. Data analysis will be largely descriptive and focus on recruitment, attendance and data provision rates. The findings will inform the sample size required for a definitive trial. A detailed process evaluation using qualitative and quantitative methods will be conducted with a variety of stakeholders (i.e. pupils, parents, teachers and peer-supporter trainers) to identify areas of success and necessary improvements prior to proceeding to a definitive trial.

**Discussion:**

The study will provide the information necessary to design a fully powered trial should PLAN-A demonstrate evidence of promise. This paper describes an update to the protocol for the PLAN-A feasibility cluster randomised controlled trial related to the data-linkage component.

**Trial registration:**

ISRCTN12543546

## Introduction

The Peer-Led physical Activity iNtervention for Adolescent girls (PLAN-A) study is a feasibility cluster randomised controlled trial of a peer-led intervention which aims to increase adolescent girls’ physical activity. The PLAN-A study involves training girls in year 8 of secondary school, who have been nominated by their peers to be “peer-supporters”, to encourage, facilitate and support their friends’ physical activity. The objectives of the feasibility study are to estimate recruitment and data provision rates, examine intervention acceptability, estimate the potential effect of the intervention on increasing girls’ moderate-to-vigorous physical activity levels, estimate the sample size needed for a definitive trial and examine the consent rate of participants and data custodians for linkage of their study data to academic and health records. The original protocol was published in *Pilot and feasibility Studies* (https://pilotfeasibilitystudies.biomedcentral.com/articles/10.1186/s40814-015-0045-8) [1].

## Update to data linkage objectives

Following publication of the original protocol, the study team held discussions with the Institutional Ethics Committee, Trial Steering Committee, Trial Management Group and data linkage experts regarding the data linkage protocol. As a result of these discussions, study objective 8 has been changed and we will now qualitatively examine parents’ views regarding allowing their child’s data to be used for data linkage with academic records kept by the National Pupil Database (NPD). We will continue to examine the proportion of demographic data (full name, DOB and home postcode) collected from pupils that are of sufficient quality and completeness to link to educational attainment data held by the NPD. As asking participants and parents to consent to a hypothetical data linkage scenario was deemed to be too complex and not likely to accurately reflect responses to a real request for consent, the original proposal to seek hypothetical consent from parents, data custodians and local authorities to take part in data linkage has been changed. We will now include questions about data linkage in interviews with parents of participants. In the interviews, we will qualitatively explore parents’ understanding of data linkage, their concerns and the length of time that they would like their consent to link study data to remain in place. Parents will be asked to comment on their views on allowing their child’s study data to be linked to educational data kept by the NPD. In addition, senior school management in each school will be asked whether they would consent to study participants’ data being used for educational data linkage. The NPD administrators will be asked if they would hypothetically allow a future trial of PLAN-A to link pupil data with attainment and absence data they hold. All changes have been agreed by the Research Ethics Committee of the School for Policy Studies at the University of Bristol, the independent Trial Steering Committee and the project funder.
